# Clinically Validated XAI for Calcified Plaque Segmentation in Coronary CT Angiography

**DOI:** 10.3390/jcm15156039

**Published:** 2026-08-03

**Authors:** Julius Siaulys, Agne Paulauskaite-Taraseviciene, Antanas Jankauskas, Gintare Sakalyte, Dovydas Verikas

**Affiliations:** 1 Artificial Intelligence Centre, Faculty of Informatics, Kaunas University of Technology, LT-44249 Kaunas, Lithuania; 2 Centre of Excellence for Sustainable Living and Working (SustAInLivWork), LT-51423 Kaunas, Lithuania; 3 Institute of Cardiology, Lithuanian University of Health Sciences, LT-50161 Kaunas, Lithuania; 4 Laboratory for Automation of Cardiovascular Investigation, Lithuanian University of Health Sciences, LT-50161 Kaunas, Lithuania

**Keywords:** coronary computed tomography, calcified plaques, explainable AI, segmentation

## Abstract

**Background**: Accurate segmentation of calcified plaques in coronary computed tomography angiography (CCTA) images is critical for reliable assessment of coronary atherosclerotic burden and for supporting interpretation of luminal stenosis, yet it remains a challenging task due to annotation inconsistencies, blooming artifacts and low contrast at lesion boundaries. These limitations may affect both automated model performance and the clinical trustworthiness of AI systems. This study explores the impact of annotation refinement on segmentation performance and model explainability, as well as the influence of representation learning on explanation quality. **Methods**: We trained a deep convolutional neural network to segment calcified plaques in coronary arteries using a dataset of expert-labeled CT slices. Initial training on radiologist-provided annotations yielded suboptimal results. To address this, annotations were manually revised and validated by radiologists. In addition to a standard ImageNet-pretrained model, we evaluated a self-supervised representation learning approach using DINOv2. Grad-CAM was used to generate visual explanations for model predictions before and after annotation refinement. **Results**: Models trained with refined annotations achieved notably improved segmentation accuracy, with clearer delineation of calcified regions. Grad-CAM localization analysis demonstrated improved concentration of model attention within plaque and vessel regions. Furthermore, models incorporating DINOv2 representations produced more spatially coherent attention maps, with improved anatomical localization consistent with coronary vessel regions and reduced off-target activations, as qualitatively confirmed by expert radiologists. **Conclusions**: Our findings emphasize the importance of high-quality, validated annotations in developing accurate and interpretable AI models for medical imaging. In addition, the results suggest that representation learning influences the reliability and clinical relevance of explainability outputs. The combination of manual annotation refinement, expert validation, and improved feature representations provides a practical workflow for human-in-the-loop AI development in cardiovascular imaging. This study demonstrates that annotation quality is a critical and often underestimated determinant of XAI reliability, and suggests that explainability methods can serve as feedback tools for iterative, clinician-guided dataset curation in cardiovascular imaging.

## 1. Introduction

Cardiovascular diseases remain the leading cause of death globally, with coronary artery disease (CAD) accounting for a substantial proportion of morbidity and mortality. Coronary computed tomography angiography (CCTA) has emerged as a critical non-invasive imaging modality for assessment of coronary atherosclerosis, enabling visualization of both luminal stenosis and plaque composition. Among plaque subtypes, calcified plaques represent an established marker of cumulative atherosclerotic burden and are strongly associated with cardiovascular risk. Accurate identification and delineation of calcified plaques in CCTA are clinically relevant for several reasons. First, plaque burden contributes to overall risk stratification and may influence preventive therapeutic decisions [1]. Second, calcified plaques can significantly affect the visual and quantitative assessment of luminal narrowing due to blooming artifacts, potentially leading to overestimation of stenosis severity [2]. Third, reproducible plaque quantification is essential for longitudinal follow-up, particularly in patients undergoing medical therapy aimed at stabilizing or modifying plaque characteristics [3]. Despite its clinical importance, manual segmentation and evaluation of calcified plaques remain time-consuming and subject to substantial interobserver variability. Small or borderline calcifications, especially in distal vessel segments, may be inconsistently annotated. Furthermore, plaque boundaries are often ambiguous, leading to heterogeneity in ground truth labeling. The rapid advancement of deep learning has fundamentally transformed medical image analysis, enabling healthcare professionals to achieve higher levels of diagnostic accuracy, reproducibility, and automation in disease detection and prognosis. Automated processing of medical images encompasses a broad spectrum of tasks, including image reconstruction, retrieval, deep-learning-based segmentation, quantitative analysis, and visualization [4,5,6,7]. Medical image computing has evolved from traditional image processing techniques toward integrated frameworks that combine computer vision, pattern recognition, data mining, and machine learning methodologies [8]. Beyond general medical image analysis, deep learning has substantially advanced the automation of cardiovascular imaging tasks, particularly the segmentation of coronary vessels [9,10,11,12] and atherosclerotic plaques [13,14,15], which are essential for accurate diagnosis, risk stratification, and prognosis assessment.

Although deep learning–based techniques have demonstrated promising performance, two major barriers continue to limit their routine clinical adoption: reliance on high-quality annotations and insufficient model transparency. Inaccurate or inconsistent labels—often present in large-scale datasets due to the difficulty of delineating small or ambiguous structures—can substantially compromise model learning and generalization [16,17]. Consequently, there has been growing interest in developing methods and techniques that reduce reliance on time-consuming and highly precise expert segmentation, thereby facilitating the creation of sufficiently large and diverse training datasets [18,19,20]. Furthermore, the black-box nature of deep learning systems raises legitimate concerns among clinicians regarding interpretability and trust, particularly in high-stakes diagnostic contexts [21,22,23]. Addressing these issues is crucial for translating promising research into routine clinical practice.

Recent advances in self-supervised learning have introduced foundation models such as DINOv2, which learn structure-aware visual representations from large-scale unlabeled data. These models have demonstrated strong generalization capabilities and improved robustness in low-data and noisy-label settings [24]. However, their role in medical image segmentation—particularly in relation to annotation quality and explainability—remains insufficiently explored.

While several studies have addressed plaque segmentation using convolutional neural network (CNN)–based models [25], many overlook the critical role of annotation quality and rarely incorporate iterative correction or systematic validation by clinical experts. In practice, manual plaque delineation in coronary CT angiography is highly sensitive to observer interpretation, lesion size, and image artifacts, leading to label inconsistency that can substantially affect model learning and generalizability [26,27]. Furthermore, although explainable artificial intelligence (XAI) methods such as Gradient-weighted Class Activation Mapping (Grad-CAM) have been increasingly employed to visualize model attention, their reliability remains uncertain when models are trained on noisy or suboptimal annotations [28,29]. Importantly, existing plaque segmentation studies typically treat annotations as fixed ground truth and use explainability primarily as a post-hoc visualization tool rather than as a mechanism to identify annotation bias or clinically implausible model behavior. The interaction between annotation quality, learned representations, and explanation reliability has received limited systematic investigation [30], particularly in the context of coronary CT imaging.

The novelty of this work lies in three main aspects. First, we systematically investigate how annotation refinement influences both segmentation performance and Grad-CAM explainability outputs. Second, we evaluate DINOv2-based self-supervised representations for calcified plaque segmentation in CCTA and analyze their impact on both predictive performance and explanation quality. Third, we incorporate vessel-aware training constraints and quantitative explainability analysis to improve anatomical plausibility and assess whether model attention is concentrated within clinically relevant plaque and coronary vessel regions.

The primary clinical use case targeted in this study is automated assessment of calcified plaque burden to support cardiovascular risk stratification, longitudinal monitoring of atherosclerotic disease progression, and decision support in preventive cardiology. The proposed framework is not intended to replace clinical interpretation, but rather to provide consistent and reproducible plaque quantification that may assist clinicians in evaluating coronary atherosclerotic burden.

Accordingly, this study pursues four principal objectives:

- To quantitatively evaluate the impact of annotation refinement on segmentation accuracy and lesion-level detection performance.
- To quantitatively and qualitatively assess how annotation refinement influences the spatial coherence, anatomical plausibility, and clinical relevance of Grad-CAM explanation maps.
- To investigate the effect of representation learning, including DINOv2-based features, on segmentation robustness and explainability.
- To analyze the interaction between annotation quality and learned representations in shaping clinically meaningful and interpretable model outputs.

We hypothesize that improved annotation quality enhances both segmentation accuracy and explanation reliability, and that self-supervised representations may further improve robustness to annotation variability while producing more anatomically coherent attention maps.

## 2. Materials and Methods

### 2.1. Dataset Description

In this study, we present a refined semantic segmentation framework for the detection of calcified plaques in coronary arteries using computed tomography (CT). Calcified plaque segmentation represents a clinically relevant yet technically challenging task due to subtle lesion boundaries, blooming artifacts, inter-observer variability, and inconsistencies in manual delineation. An initial segmentation model trained on radiologist-provided annotations demonstrated limited generalization performance, which we attributed primarily to imprecision and heterogeneity in the ground-truth labels. To address this limitation, we implemented a structured annotation refinement protocol. Using domain-specific knowledge of coronary anatomy and standardized visual assessment criteria, initial plaque annotations were systematically reviewed and manually corrected to ensure consistent boundary delineation. The refined labels were subsequently validated by experienced radiologists to confirm anatomical plausibility and clinical correctness. This human-in-the-loop refinement process aimed to reduce label noise and improve the quality of supervision during model training.

This retrospective study was conducted using a proprietary dataset of coronary computed tomography angiography (CCTA) images acquired during routine clinical practice. The dataset comprised 47 patients and included the three major coronary arteries: the left anterior descending (LAD), left circumflex (LCX), and right coronary artery (RCA). For each vessel, an average of eight axial slices were annotated. In total, 1259 CT slices were included, yielding 1259 coronary artery annotations and 1532 distinct calcified plaque instances. The annotated images corresponded to all vessel-specific slices generated by the coronary vessel extraction software. No additional manual slice selection was performed by the authors, and images were not selected on the basis of plaque burden, lesion severity, or visual appearance. Consequently, the dataset reflects the complete output of the vessel extraction workflow rather than a manually curated subset of cases.

All imaging data were anonymized prior to analysis in accordance with institutional data protection policies. Initial plaque annotations were performed by board-certified radiologists using standard clinical interpretation software. The refined annotations were independently reviewed and validated by the different two senior radiologists with more than 10 years of experience in cardiovascular imaging. Visual inspection of the initial labels revealed inconsistencies in plaque boundary delineation, particularly for small or borderline calcifications and regions affected by blooming artifacts, consistent with known challenges in CCTA interpretation.

To improve annotation quality, a structured refinement process was conducted by a researcher with domain-specific expertise in cardiovascular imaging. Annotation corrections were guided by standardized visual assessment criteria, including continuity along the vessel centerline, exclusion of isolated high-intensity noise, and consistent boundary handling of blooming artifacts. Where ambiguity was present, conservative delineation strategies were applied to reduce inclusion of non-coronary structures.

The refined annotations were subsequently reviewed and independently validated by two senior radiologists with more than 10 years of experience in cardiovascular imaging, who confirmed their anatomical plausibility and clinical correctness. In cases of disagreement, annotations were jointly reviewed and a consensus was reached. This multi-step validation process was designed to reduce label noise and ensure consistency across the dataset.

**Figure 1:**
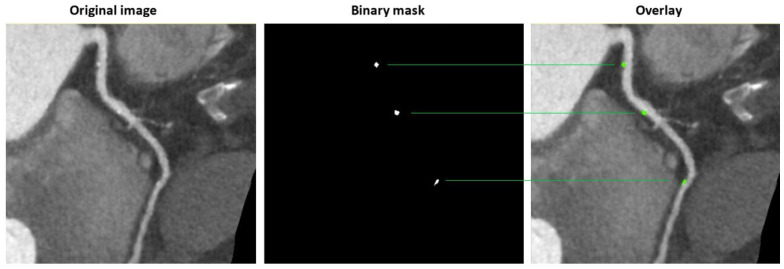
Example of right coronary artery (RCA) annotation with delineated calcified plaques.

An example of annotated coronary artery slices with delineated calcified plaques is shown in Figure 1.

### 2.2. Preprocessing Pipeline

All input CT images were resized to a fixed spatial resolution of 512×512 pixels to standardize the input dimensions for the segmentation network. To incorporate anatomical context, a multi-channel input representation was constructed.

The first channel consisted of the original CT image. The second channel corresponded to a binary coronary vessel mask, providing explicit structural information about vessel boundaries. The third channel replicated the original CT image to maintain compatibility with pretrained encoders while preserving intensity information. Coronary vessel masks were generated automatically using a separately trained vessel-segmentation model and were provided as part of the preprocessing pipeline. These masks were used during both training and inference to provide anatomical context and to support the vessel-aware loss function.

Prior to channel construction, intensity values were clipped using a plaque-specific Hounsfield Unit (HU) thresholding strategy. The clipping range was determined from the median HU values observed within the annotated plaque regions and was used to improve plaque boundary delineation and reduce the influence of surrounding tissues. The resulting multi-channel input was normalized using the ImageNet mean [0.485,0.456,0.406] and standard deviation [0.229,0.224,0.225] to enable effective transfer learning from pretrained encoder weights.

Segmentation masks were loaded as single-channel grayscale images with discrete label values and converted to class indices spanning [0, NUM_CLASSES-1]. Nearest-neighbor interpolation was applied during resizing to preserve label integrity.

To improve generalization and reduce overfitting, data augmentation techniques including random rotations, scaling, and elastic deformations were applied during training. All preprocessing and augmentation steps were implemented using the PyTorch 2.10.0 and torchvision frameworks.

### 2.3. Model Architecture and Training

Following annotation validation, the segmentation model was retrained using the refined labels, and a comparative performance analysis was conducted. In addition, we evaluated the influence of representation learning by incorporating a self-supervised DINOv2-based model alongside a standard ImageNet-pretrained baseline. In parallel, Grad-CAM was integrated to generate post-hoc visual explanations of model predictions, enabling assessment of whether network attention aligned with anatomically and clinically meaningful plaque regions. Calcified plaque segmentation was performed using a two-dimensional semantic segmentation network based on an encoder–decoder architecture. To investigate the impact of representation learning, two model variants were implemented:

- **Supervised baseline:** An encoder initialized with ImageNet-pretrained ResNet101 weights.
- **Self-supervised model:** An encoder initialized using DINOv2 pretrained weights, providing structure-aware feature representations learned from large-scale unlabeled data.

ResNet101 was selected as the baseline architecture because of its extensive use in medical image segmentation and transfer-learning applications. Its widespread adoption in the literature provides a strong and reproducible benchmark for comparison with DINOv2-based representations.

**Figure 2:**
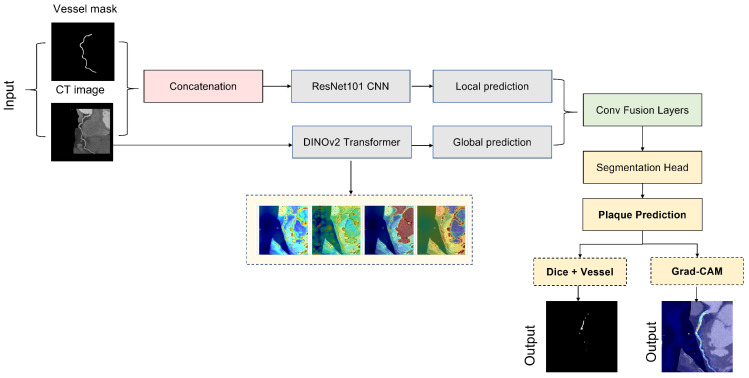
The solution pipeline based on DINOv2 architecture.

Both models shared an identical decoder architecture and were trained under the same conditions to ensure a fair comparison. The models processed individual axial CT slices of size 512×512 pixels and produced binary output masks indicating the presence or absence of calcified plaque (see Figure 2).

**Figure 3:**
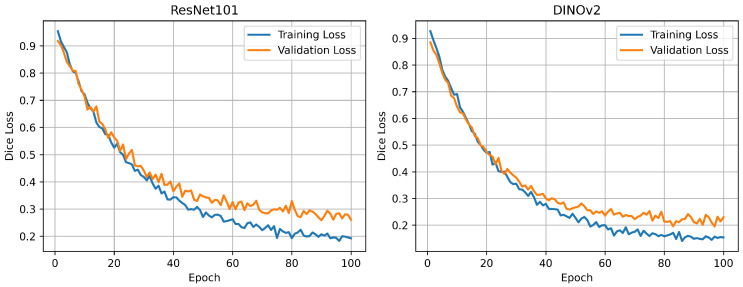
Training and validation loss curves.

Model training was conducted using PyTorch with the Adam optimizer, an initial learning rate of 0.001, a batch size of 32, and 100 training epochs. A Dice-based loss function was employed to directly optimize spatial overlap between predicted and reference segmentations. The training process is illustrated in Figure 3.

The dataset was split into training (75%), validation (15%), and testing (10%) subsets at the patient level using a fixed random seed to ensure reproducibility and to prevent data leakage. This resulted in 35 patients in the training set, 7 patients in the validation set, and 5 patients in the test set. A softmax activation was applied during training, where class 0 represented background and class 1 represented calcified plaque. In addition, a vessel-aware constraint was incorporated into the training objective to penalize predictions outside coronary vessel regions, thereby reducing anatomically implausible false positives.

The training objective was defined as:

$$L=L_{Dice}+\lambda \cdot L_{vessel}$$

where $L_{Dice}$ denotes the Dice loss and $L_{vessel}$ penalizes predicted plaque probabilities outside vessel regions:

$$L_{vessel}=\frac{1}{N}\sum_{i}\left(1-V_{i}\right)\cdot P_{i}$$

where $V_{i}$ is the vessel mask and $P_{i}$ is the predicted plaque probability.

The weighting parameter λ was empirically set to 0.1 based on validation performance.

### 2.4. Explainability Analysis with Grad-CAM

To assess model interpretability, Gradient-weighted Class Activation Mapping (Grad-CAM) was applied as a post-hoc explainability method. Grad-CAM visualizations were generated using the final segmentation layer of the decoder as the target layer. For the segmentation task, the target class corresponded to the calcified plaque class (Class 1), and gradients were computed with respect to the predicted plaque probability map. Preliminary experiments were performed using multiple decoder layers; however, the final segmentation layer was selected because it provided the most spatially precise localization and strongest correspondence with annotated plaque regions. Gradients corresponding to the calcified plaque class were back-propagated to selected convolutional layers to generate class-specific attention maps highlighting regions that most strongly influenced the model’s predictions.

Grad-CAM analysis was performed for both the ImageNet-pretrained baseline model and the DINOv2-based model to evaluate the influence of representation learning on explanation quality. The resulting heatmaps were upsampled to the original image resolution and overlaid on the corresponding CT slices.

These visualizations enabled qualitative assessment of whether model attention aligned with anatomically and clinically plausible plaque regions, as well as comparison of spatial coherence and localization between the two representation learning scenarios. Particular attention was given to identifying off-target activations and assessing whether model explanations were confined to coronary vessel structures.

To provide a quantitative assessment of explanation quality, the fraction of normalized Grad-CAM energy located within anatomically relevant regions was calculated. For each Grad-CAM heatmap, values were normalized to the range [0, 1]. The Grad-CAM energy within a given mask was defined as the sum of normalized heatmap values inside the mask divided by the total heatmap energy:

$$E_{M}=\frac{\sum_{i\in M}H_{i}}{\sum_{i}H_{i}}$$

where $H_{i}$ denotes the normalized Grad-CAM value at pixel *i*, and *M* represents either the ground-truth plaque mask or the coronary vessel mask. Higher values indicate that model attention is more concentrated within clinically relevant anatomical regions.

### 2.5. Evaluation by Clinical Experts

Segmentation outputs and corresponding Grad-CAM overlays were independently reviewed by two radiologists with over 10 years of experience in cardiovascular imaging. To assess annotation reliability, two senior radiologists independently annotated a randomly selected subset of previously unseen cases. Inter-observer agreement was calculated using the Dice similarity coefficient. Intra-observer agreement was evaluated by repeated annotation of the same subset after a washout period of 3 weeks. Following completion of the annotation task, the experts reviewed the corresponding segmentation outputs and Grad-CAM visualizations generated by the models. Each expert assessed the anatomical plausibility and clinical relevance of the predicted plaque regions and explanation maps, with particular emphasis on the spatial alignment of attention maps with calcified plaque locations and their confinement within coronary vessel structures. Comparative assessment between the ResNet101-based and DINOv2-based models indicated that the latter more consistently produced anatomically coherent and clinically meaningful attention maps, with reduced off-target activations. Discrepancies in interpretation were discussed jointly to reach consensus, and expert feedback was used to contextualize false positive and false negative predictions as well as to inform future annotation refinement strategies.

### 2.6. Statistical Analysis

Statistical analyses were performed using Python 3.11. Performance metrics are reported as median values across the evaluated samples. Differences between the ResNet101-based and DINOv2-based models were assessed using the Wilcoxon signed-rank test. A *p*-value below 0.05 was considered statistically significant.

## 3. Results

### 3.1. Impact of Annotation Refinement

To directly evaluate the effect of annotation refinement, segmentation performance was compared between models trained using the original radiologist annotations and the refined annotation set. Annotation refinement resulted in substantial improvements across all evaluated metrics (Table 1). The Dice coefficient increased from 0.611 to 0.732 (*p* < 0.001), while precision improved from 0.648 to 0.762 (*p* = 0.002) and recall from 0.592 to 0.718 (*p* < 0.001). A corresponding reduction in Hausdorff distance was also observed, indicating improved boundary localization.

### 3.2. Observer Agreement Analysis

Inter-observer and intra-observer agreement were evaluated to assess the consistency of plaque annotations. The median inter-observer Dice coefficient was 0.82, indicating good agreement between the two radiologists. The median intra-observer Dice coefficient was 0.84, showing that repeated annotations by the same observer were highly consistent.

### 3.3. ROC Analysis

**Figure 4:**
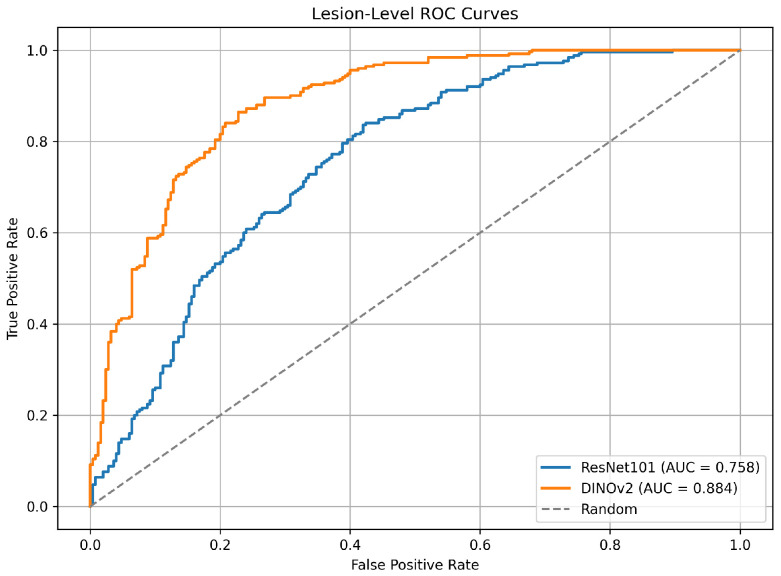
Lesion-level receiver operating characteristic (ROC) curves for the ResNet101-based and DINOv2-based models.

Lesion-level ROC analysis was performed to assess the ability of both models to discriminate between true plaque and non-plaque findings. The ResNet101-based model achieved an AUC of 0.758, whereas the DINOv2-based model achieved an AUC of 0.884. The corresponding ROC curves are shown in Figure 4.

The higher AUC achieved by the DINOv2-based model is consistent with the lesion-level performance metrics presented in Section 3.5.

### 3.4. Pixel-Wise Segmentation Metrics

Segmentation performance was evaluated using pixel-wise metrics on the training, validation, and test sets. Table 2 summarizes the Dice Similarity Coefficient, precision, recall, false negative rate (FNR), and Hausdorff distance for calcified plaque segmentation for both the ResNet101-based and DINOv2-based models.

The DINOv2-based model achieved a median Dice score of 0.7322 on the test set, compared to 0.7104 for the ResNet101-based model. Precision and recall values for the DINOv2 model were 0.7615 and 0.7181, respectively, while the ResNet101-based model achieved a precision of 0.7322 and a recall of 0.7097. The DINOv2 model also demonstrated a lower false negative rate (0.2819 vs. 0.3121) and improved boundary localization, as reflected by a reduced Hausdorff distance (3.5811 vs. 3.9873).

Overall, segmentation performance was comparable between the two models, although the DINOv2-based model demonstrated statistically significant improvements in Dice score, Hausdorff distance, specificity, and accuracy, suggesting that the use of self-supervised representations preserves predictive accuracy. These results indicate that improved representation learning can be achieved without compromising segmentation performance.

**Figure 5:**
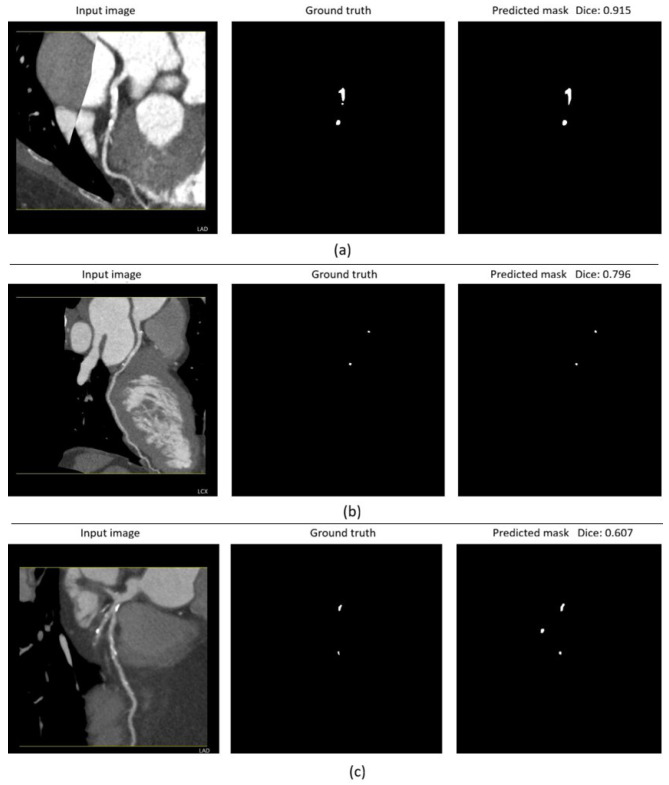
Examples of segmentation results showing input CT images, ground truth annotations, and predicted masks for calcified plaques, with Dice scores indicating segmentation quality: (**a**) Dice = 0.915; (**b**) 0.796; (**c**) 0.607.

This Figure 5 shows coronary artery calcification segmentation results across three CT cases, each displaying an input image, ground truth mask, and predicted mask with its Dice score. In panel (a) Dice value of 0.915 shows excellent segmentation, where the predicted mask closely matches the ground truth calcification regions; (b) shows good performance in very small and sparse calcification, although there are minor discrepancies; (c) The ground-truth annotation contains two calcified plaques, but the predicted mask shows 3 plaques, meaning the model generated a false positive (one extra calcification that does not exist); therefore, the Dice score is equal to 0.607. In some cases, the model completely fails to overlap with the ground truth, resulting in a Dice score of 0.00. Cases with Dice scores of 0.00 were typically associated with very small or fragmented calcifications, lesions located near the detection threshold, or localization errors where the predicted region did not overlap with the reference annotation. Because the Dice coefficient requires spatial overlap between the predicted and ground-truth masks, even small localization discrepancies may result in a score of zero despite partial identification of clinically relevant structures.

Overall, the model performs well on larger/clearer lesions but struggles with small, fragmented calcifications, which is a common challenge in coronary calcium scoring tasks.

### 3.5. Lesion-Level Detection Performance

To evaluate clinically meaningful detection capability, lesion-level metrics were computed by treating each distinct calcified plaque instance as a detection target. Table 3 summarizes lesion counts and detection statistics across data splits for both the ResNet101-based and DINOv2-based models.

The test set consisted of 5 patients, 120 annotated slices, and 143 calcified plaque instances.

Across both models, the average number of predicted lesions per slice closely matched ground truth lesion counts, indicating that neither approach systematically over- or under-detected plaques. On the validation and test sets, the DINOv2-based model demonstrated lesion count estimates comparable to the ResNet101-based model (e.g., validation: 2.0935 vs. 2.0535; test: 2.3382 vs. 2.4231 predicted lesions per slice).

Lesion-level precision, recall, and F1-score remained consistently high for both models on the test set. The DINOv2-based model achieved lesion-level precision, recall, and F1-score of 0.7233, 0.7730, and 0.7326, respectively, compared to 0.7199, 0.7636, and 0.7183 for the ResNet101-based model. Overall, differences between the two models were modest, although the DINOv2-based model achieved a statistically significant improvement in lesion-level F1 score, indicating that the use of self-supervised representations preserves lesion detection performance.

While the Dice coefficient provides a measure of segmentation accuracy in terms of pixel-wise overlap, lesion-level metrics assess the clinical relevance of model predictions by quantifying detection performance. High lesion precision indicates that few false plaques are predicted, whereas high lesion recall reflects the model’s sensitivity in identifying all true lesions. This distinction is especially important in clinical contexts where missing a plaque (false negative) or predicting a non-existent one (false positive) can impact downstream decision-making.

For lesion-level evaluation, a predicted lesion was considered a true positive if its intersection-over-union (IoU) with a ground-truth lesion exceeded 0.5. Predicted lesions without a matching ground-truth lesion were counted as false positives, while ground-truth lesions without a corresponding prediction were counted as false negatives. In cases involving merged or split lesions, matching was assigned based on the highest IoU value.

### 3.6. False Positive Rate in Plaque-Free Slices

False positive predictions were analyzed in slices without annotated calcified plaques. Table 4 reports false positive rates for healthy cases across training, validation, and test splits for the ResNet101-based and DINOv2-based models.

False positive rates remained below 13% across all subsets, indicating stable discrimination between plaque-containing and plaque-free slices. The ResNet101-based model demonstrated slightly higher false positive rates during training, while both models showed comparable behavior on validation and test sets. Qualitative inspection revealed that the DINOv2-based model produced fewer anatomically implausible activations, particularly outside coronary vessel regions.

### 3.7. Qualitative Analysis of False Positives

To better characterize model error patterns and assess their clinical implications, a detailed review of all false-positive predictions was performed in the validation and test sets. Three predominant categories of false positives were identified, each with distinct methodological underpinnings:

1. **Predictions outside the boundaries of coronary vessels**
  The most frequently observed error category consisted of predictions located outside of the annotated regions of the coronary vessels. These activations are most likely related to adjacent anatomical structures (pericardial adipose tissue, myocardial tissue, or calcified non-coronary structures) as well as imaging artifacts that are misclassified as calcification. This category accounted for the majority of false positives across both evaluation sets
  - Test Set: 3 out of 8 false positives
  - Validation Set: 2 out of 8 false positives
  This pattern of errors suggests that additional anatomical constraints may improve localization of model predictions. Therefore, we built in a training penalty that discourages the model from flagging regions outside the vessel boundaries. In practice, the DINOv2-based model responded well to this constraint, while the ResNet101-based model tended to spread its activations more broadly, picking up signals in areas unlikely to contain plaque.
2. **Small or borderline lesions**
  These regions correspond to very small or borderline calcifications, potentially representing early-stage or clinically insignificant deposits near the detection threshold.
  - Test Set: 3 out of 8 false positives
  - Validation Set: 2 out of 8 false positives
  These cases remain challenging to assess, even for experienced radiologists. Radiologists often disagree on whether such a small object is a genuine atherosclerotic plaque or merely image noise. Therefore, these predictions should not necessarily be interpreted as unequivocal model errors, they reflect a broader problem. These findings illustrate the continuous nature of plaque development and the limitations of binary annotation schemes. In the future, it might be worth allowing annotators to flag ambiguous cases rather than forcing them to assign them to categories of “yes” or “no”.
3. **Missed annotations**
  These predictions aligned with anatomically plausible plaque regions that were not labeled in the ground truth, highlighting residual annotation variability.
  - Test Set: 0 out of 8 false positives
  - Validation Set: 2 out of 8 false positives

These cases suggest residual variability in annotation within the refined dataset and are consistent with the known challenges of exhaustive plaque delineation in CCTA, particularly in distal vessel segments and regions affected by blooming artifacts. These findings may reflect residual annotation variability and potential omission of subtle plaque regions. This observation confirms that it is worthwhile to use model predictions (i.e., explainability maps) as a feedback mechanism to help identify potentially missed features during dataset reprocessing.

Qualitative comparison of model behavior indicated that the DINOv2-based model produced more spatially constrained predictions, with fewer activations extending beyond vessel regions, whereas the ResNet101-based model was more prone to diffuse activations in surrounding tissue.

Overall, the small number of false positives in both evaluation sets shows that the model generalizes well to new unseen cases. The observed error patterns were associated with identifiable anatomical and annotation-related factors. Predictions outside the vessel boundaries suggest that the model could benefit from stronger anatomical guidance. Borderline lesions and missed annotations are less about model failure and more about the difficulty of the task itself. Certain plaque regions remain inherently challenging to delineate, and residual uncertainty in the reference annotations may persist despite expert review.

**Figure 6:**
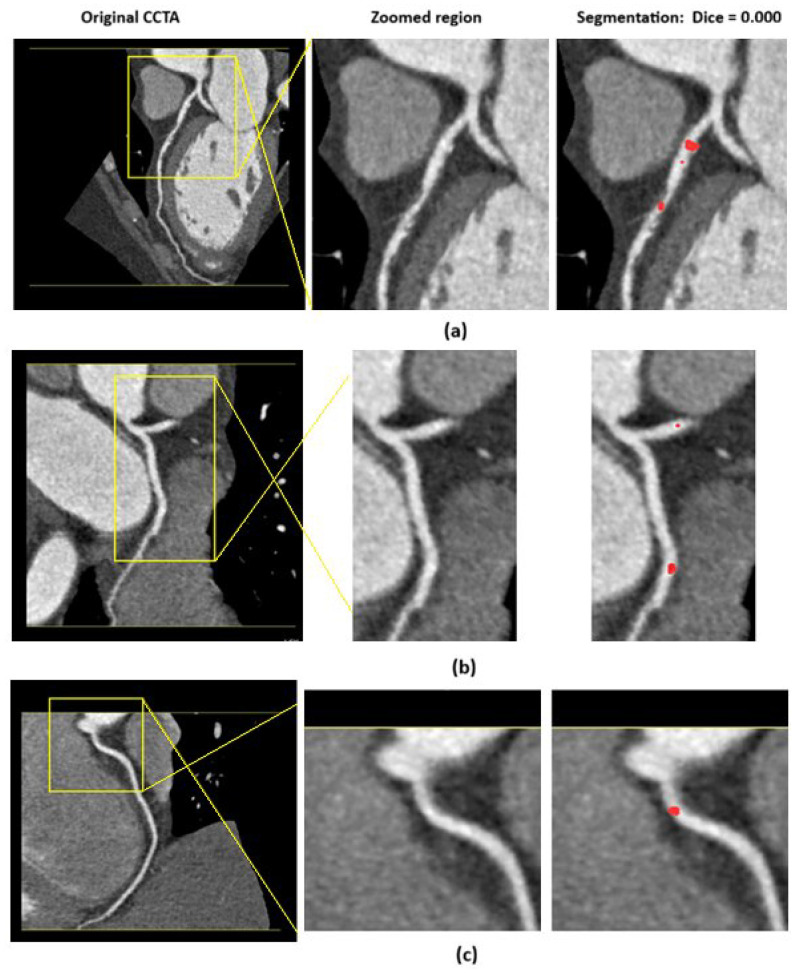
Examples of unannotated coronary calcifications in CCTA images identified by the AI model. (**a**) Two larger calcifications and one insignificant calcification. (**b**) Two very small clinically irrelevant calcifications and one medium sized calcification identified by the model. (**c**) A single calcification detected by the AI model that was missed in the expert-defined mask. Yellow lines indicate the zoomed-in region shown for improved clarity. Red dots mark calcification centers that were identified by the model.

Figure 6 provides some instances where AI-identified calcified regions were overlooked by human annotators, raising questions about the completeness of expert-defined ground truth masks.

### 3.8. Grad-CAM Explainability Analysis

Grad-CAM explanation maps were generated to assess whether model attention aligned with anatomically and clinically relevant calcified plaque regions. Based on preliminary analysis, explanations derived from the final segmentation layer provided the most spatially precise and clinically meaningful visualizations; therefore, subsequent analysis focuses on Grad-CAM maps obtained from the segmentation head.

A qualitative comparison between the ResNet101-based and DINOv2-based models revealed distinct differences in attention patterns. The ResNet101 model exhibited relatively diffuse and fragmented attention, with activations frequently extending beyond coronary vessel boundaries into surrounding tissue. While some activations overlapped with plaque regions, spatial localization was often imprecise. In contrast, the DINOv2-based model generated more spatially coherent and sharply localized attention maps that closely aligned with annotated calcified plaques. Model activations were more consistently confined to coronary vessel regions, indicating improved anatomical awareness and reduced off-target focus. These differences were particularly evident in regions with complex structure or subtle calcifications, where the DINOv2 model demonstrated clearer delineation of relevant features and stronger correspondence with clinically meaningful areas.

Overall, Grad-CAM analysis suggests that representation learning influences not only segmentation performance but also the reliability and interpretability of model explanations. The DINOv2-based model provides more anatomically consistent and clinically plausible visualizations, supporting its potential for interpretable AI-driven cardiovascular imaging.

**Figure 7:**
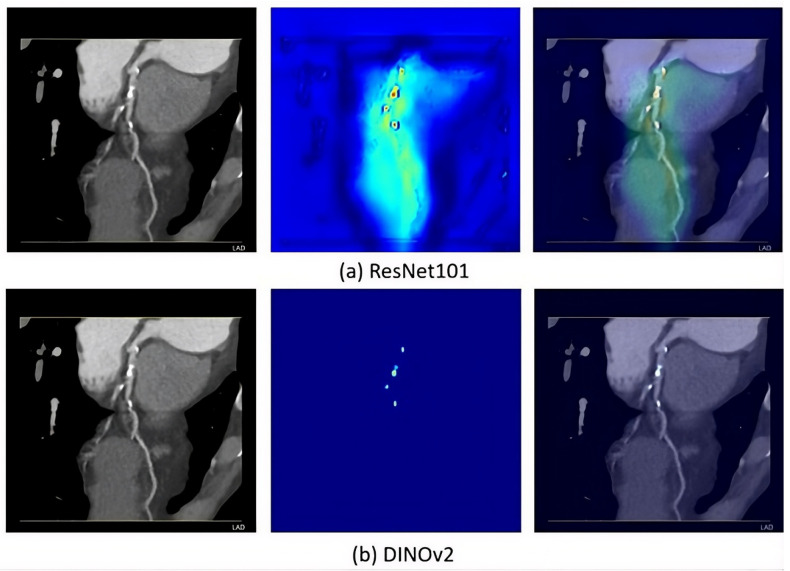
Comparison of Grad-CAM explanation maps generated by the (**a**) ResNet101-based model and (**b**) DINOv2-based model. Each triplet shows the input CCTA image, ground-truth plaque annotation, and corresponding Grad-CAM visualization. Warmer colors (red/yellow) indicate regions with a stronger contribution to the model prediction, whereas cooler colors (blue) indicate weaker influence. The DINOv2-based model demonstrates more spatially coherent activations concentrated within plaque and vessel regions, while the ResNet101-based model exhibits more diffuse activations extending into surrounding tissue.

In Figure 7 each triplet shows the input CT image, ground truth annotation, and corresponding Grad-CAM visualization. While both models achieve comparable segmentation results, the DINOv2-based model demonstrates more spatially coherent and anatomically aligned attention patterns, whereas the ResNet101 model exhibits more diffuse and less localized activations.

### 3.9. Quantitative Grad-CAM Localization Analysis

To quantitatively assess explanation localization, the fraction of Grad-CAM energy contained within ground-truth plaque masks and vessel masks was calculated. The DINOv2-based model demonstrated a higher proportion of Grad-CAM energy within both plaque and vessel regions compared with the ResNet101-based model, indicating improved anatomical localization of model attention. These findings are consistent with the qualitative observations presented in the Grad-CAM analysis and support the interpretation that DINOv2 representations produce more spatially coherent explanations. Results can be seen in Table 5.

Approximately 68% of Grad-CAM energy was concentrated within plaque regions for the DINOv2-based model, compared with 43% for the ResNet101-based model. Similarly, 91% of Grad-CAM energy was concentrated within coronary vessel regions for the DINOv2-based model, compared with 64% for the ResNet101-based model.

## 4. Discussion

In this study, we investigated how annotation refinement and representation learning influence both segmentation performance and explainability in a deep learning model for calcified plaque segmentation in coronary CT angiography (CCTA). Our results demonstrate that manual annotation correction, when validated by experienced radiologists, substantially improves segmentation accuracy while producing explainability outputs that are more anatomically aligned and clinically meaningful. Furthermore, the integration of self-supervised representations through a DINOv2-based architecture enabled more spatially coherent and interpretable model behavior without compromising predictive performance.

Accurate identification of calcified plaques in CCTA is not merely a technical segmentation task. In clinical practice, the presence, extent, and distribution of coronary calcification contribute to assessment of atherosclerotic burden and may influence decisions regarding preventive pharmacotherapy, risk factor management, and further diagnostic testing. Extensive calcification can also impair visual interpretation of luminal narrowing due to blooming artifacts, potentially leading to overestimation of stenosis severity. In this context, inconsistent or imprecise plaque delineation may propagate downstream errors, particularly when quantitative or semi-quantitative analyses are applied.

The initial segmentation model trained on unrefined radiologist annotations exhibited limited generalization, which we attribute primarily to inter-observer variability and ambiguous lesion boundaries. This highlights a critical challenge in clinical AI: models trained on imperfect labels may inherit and amplify variability present in routine reporting. By applying a structured annotation refinement protocol and validating corrected labels through expert review, we provided higher-quality supervision during training. This resulted in significantly improved Dice scores (0.611 to 0.732, *p* < 0.001) and lower false negative rates (0.408 to 0.282, *p* < 0.001), together with more stable lesion-level detection performance. The observer agreement analysis further indicated good annotation consistency, supporting the reliability of the refined reference labels used for training and evaluation.

Importantly, annotation refinement also had a direct impact on model explainability. Grad-CAM heatmaps generated from models trained on refined annotations showed stronger spatial correspondence with true plaque locations. In contrast, models trained on noisier labels produced more diffuse and less coherent attention patterns, despite similar architectural configurations. These findings indicate that the reliability of explainability methods is fundamentally dependent on the quality of training data, and that interpretation of model explanations must be contextualized by annotation integrity.

Beyond annotation quality, representation learning played a key role in shaping model behavior. The DINOv2-based model produced more spatially coherent and anatomically constrained attention maps compared to the ResNet101 baseline, with reduced off-target activations outside coronary vessel regions. This effect was further reinforced by the incorporation of anatomical priors through multi-channel inputs and a vessel-aware penalty term during training, which discouraged predictions in anatomically implausible regions. Together, these components improved both segmentation outputs and the clinical plausibility of model explanations, highlighting the importance of combining data quality with representation design. These observations were further supported by the quantitative Grad-CAM localization analysis, where approximately 68% of Grad-CAM energy was concentrated within plaque regions for the DINOv2-based model compared with 43% for the ResNet101-based model. Similarly, 91% of Grad-CAM energy was concentrated within coronary vessel regions for the DINOv2-based model compared with 64% for the ResNet101-based model, indicating improved anatomical localization of model attention.

While accurate calcified plaque segmentation represents a necessary foundation, plaque localization alone is insufficient to determine functional stenosis severity or ischemic relevance. Clinical decision-making typically integrates plaque characteristics with vessel lumen dimensions, hemodynamic significance, and patient-level risk factors. Integrating plaque segmentation with coronary artery and lumen segmentation would enable estimation of luminal narrowing and provide more clinically actionable insights. These findings support the development of hybrid AI systems that combine image-based learning with anatomical and physiological constraints, particularly in coronary CT imaging where artifacts and subtle disease patterns introduce uncertainty.

Additionally, detailed demographic, clinical, and acquisition protocol information was not available for retrospective analysis. Although the dataset was derived from routine clinical CCTA examinations and included a heterogeneous patient population, the absence of these descriptors limits further subgroup analyses and should be addressed in future studies.

Several limitations should be acknowledged. The dataset size and single-center nature may limit generalizability, despite rigorous expert validation of annotations. External validation on multicenter cohorts would be necessary to assess robustness across different scanners, acquisition protocols, and patient populations. Additionally, while Grad-CAM provided useful qualitative insights, the quantitative reliability and clinical validity of explainability methods in medical imaging remain an open research question. Future work should explore objective metrics for explanation quality and investigate whether explainability signals can be systematically integrated into annotation refinement or training processes.

Importantly, explainability methods should not be interpreted as mechanisms for directly improving model performance but rather as tools for understanding model behavior and supporting transparency of AI systems [5]. Although Grad-CAM was selected because of its widespread use in medical imaging applications, alternative explainability approaches such as SHAP and LIME have also been proposed and may provide complementary insights [31].

The present study has several additional limitations. First, the analysis was performed using two-dimensional axial slices and therefore does not explicitly model three-dimensional spatial continuity along the coronary vessel course. Although three-dimensional approaches benefit from additional spatial information and vessel continuity, they often require volumetric reconstruction workflows, dedicated software, and substantially greater computational resources. In collaboration with radiologists, we found that preparation of volumetric coronary datasets can be operationally demanding in routine clinical practice. The proposed 2D approach therefore prioritizes workflow simplicity, lower computational requirements, and easier future deployment while maintaining competitive segmentation performance. Second, the study focused exclusively on calcified plaques and did not evaluate mixed or non-calcified plaque subtypes, which may present different imaging characteristics and segmentation challenges. Third, the proposed framework relies on pre-computed coronary vessel masks to provide anatomical context and support vessel-aware training. Potential errors in these vessel masks were not independently evaluated and may have influenced downstream segmentation performance and explainability results.

From a clinical perspective, reliable plaque segmentation may support cardiovascular risk assessment, longitudinal monitoring of atherosclerotic burden, and preventive cardiology workflows. Recent recommendations highlight the growing role of CCTA in selected populations, including master athletes and individuals at elevated cardiovascular risk, where the identification of subclinical coronary artery disease may improve risk stratification and guide clinical decision-making [32,33].

These findings are also consistent with the growing role of cardiovascular imaging in prevention strategies, where imaging-based biomarkers increasingly complement traditional risk-factor assessment and support early identification of subclinical disease [34].

From a practical deployment perspective, false-positive predictions may occur near adjacent calcified anatomical structures, including valvular and pericardial calcifications, as well as regions affected by imaging artifacts. Consequently, the proposed model is intended as a decision-support tool rather than a replacement for expert interpretation. Integration into existing CCTA reporting workflows could enable automatic highlighting and quantification of suspicious plaque regions while maintaining radiologist oversight. Such an approach may improve reporting consistency and reduce manual workload while preserving clinical accountability.

Overall, our findings emphasize that high-quality, expert-validated annotations are fundamental not only for improving segmentation performance but also for ensuring meaningful model interpretability. By demonstrating the combined impact of annotation refinement, representation learning, and anatomical constraints, this work supports a human-in-the-loop paradigm in which data curation and explainability analysis jointly contribute to the development of clinically reliable and interpretable AI systems for cardiovascular imaging.

## 5. Conclusions

This study demonstrates that high-quality annotations are essential for developing accurate and interpretable deep learning models in medical image analysis. By manually refining calcified plaque annotations in coronary CT angiography and validating them through expert review, we achieved substantial improvements in segmentation performance and consistency of lesion detection.

Beyond annotation quality, our findings highlight the importance of representation learning and anatomical constraints in shaping both model predictions and explainability. The integration of self-supervised DINOv2 representations enabled more spatially coherent and anatomically aligned feature learning, while the incorporation of vessel-aware inputs and training constraints reduced anatomically implausible predictions. Together, these components contributed to improved localization of calcified plaques and more clinically plausible model behavior.

The use of Grad-CAM further demonstrated that annotation quality and model design directly influence the reliability of explainability outputs. Models trained on refined data and enhanced representations produced attention maps that better corresponded to true plaque regions, whereas weaker supervision and baseline representations resulted in more diffuse and less interpretable activations. Quantitative Grad-CAM localization analysis additionally showed improved concentration of model attention within plaque and coronary vessel regions for the DINOv2-based model, supporting the observed improvements in anatomical coherence. These findings suggest that explainability methods not only aid interpretation but can also serve as a valuable tool for identifying limitations in both data and model design.

From a clinical perspective, improved segmentation accuracy and anatomically consistent predictions have the potential to enhance automated plaque detection and support risk stratification workflows. By reducing false positives outside vessel structures and improving alignment with true pathology, the proposed approach moves closer to clinically reliable decision support.

Overall, this work supports a human-in-the-loop paradigm for medical AI development, in which expert-validated annotations, representation learning, and explainability are jointly leveraged to produce robust and clinically meaningful models. Future work should focus on external validation in larger, multi-center datasets, integration with lumen and vessel analysis for functional assessment, and the development of quantitative metrics for evaluating explainability in medical imaging systems.

## Figures and Tables

**Table 1:** Impact of annotation refinement on calcified plaque segmentation performance. Results are reported on the test set. Statistical significance was assessed using the Wilcoxon signed-rank test.

Metric	Original Annotations	Refined Annotations	*p*-Value
Dice	0.611	0.732	<0.001
Precision	0.648	0.762	0.002
Recall	0.592	0.718	<0.001
Specificity	0.982	0.993	0.014
Accuracy	0.941	0.980	0.008
FNR	0.408	0.282	<0.001
Hausdorff	5.812	3.581	0.003

**Table 2:** Class-wise segmentation performance metrics (Class 1: calcified plaque) for the ResNet101-based and DINOv2-based models across the training, validation, and test sets. Results are reported as median values. Statistical significance between the ResNet101-based and DINOv2-based models on the test set was assessed using the Wilcoxon signed-rank test. FNR = False Negative Rate.

Metric	ResNet101			DINOv2			*p*-Value
	Train	Validation	Test	Train	Validation	Test	
Dice	0.742	0.676	0.710	0.786	0.698	0.732	0.031
Precision	0.769	0.742	0.732	0.800	0.757	0.762	0.084
Recall	0.797	0.652	0.710	0.813	0.665	0.718	0.112
Specificity	0.994	0.993	0.992	0.996	0.994	0.993	0.045
Accuracy	0.981	0.972	0.976	0.986	0.978	0.980	0.038
FNR	0.225	0.357	0.312	0.188	0.335	0.282	0.067
Hausdorff	3.400	4.305	3.987	3.000	4.000	3.581	0.019

**Table 3:** Lesion-level Detection Metrics for Class 1 Across Data Splits for ResNet101-based and DINOv2-based Models.

Metric	ResNet101			DINOv2			*p*-Value
	Train	Valid	Test	Train	Valid	Test	
GT Lesions	2.299	2.103	2.147	2.299	2.103	2.147	–
Pred Lesions	2.054	2.054	2.423	2.097	2.094	2.338	–
TP	2.001	1.792	1.814	2.006	1.785	1.838	–
FP	0.137	0.347	0.470	0.139	0.346	0.471	–
FN	0.290	0.321	0.305	0.293	0.318	0.309	–
Lesion Precision	0.825	0.757	0.720	0.879	0.761	0.723	0.088
Lesion Recall	0.842	0.781	0.764	0.856	0.787	0.773	0.054
Lesion F1 score	0.831	0.715	0.718	0.855	0.759	0.733	0.041

**Table 4:** False Positive Summary for Healthy Cases Across Training, Validation, and Test Splits for ResNet101 and DINOv2-based Models.

Split	ResNet101			DINOv2		
	Total Samples	False Positives	FP Rate (%)	Total Samples	False Positives	FP Rate (%)
Train	448	35	7.81	448	9	2.01
Validation	90	8	8.89	90	8	8.89
Test	66	8	12.12	66	8	12.12

**Table 5:** Quantitative Grad-CAM localization analysis. Values represent the fraction of normalized Grad-CAM energy contained within the corresponding masks.

Metric	ResNet101	DINOv2
Grad-CAM energy within plaque mask	0.43	0.68
Grad-CAM energy within vessel mask	0.64	0.91

## Data Availability

The data and the code used for this study are available from the corresponding author upon reasonable request.

## Abbreviations

The following abbreviations are used in this manuscript:

CCTA	Coronary Computed Tomography Angiography
CAD	Coronary Artery Disease
CT	Computed Tomography
CNN	Convolutional Neural Network
XAI	Explainable Artificial Intelligence
Grad-CAM	Gradient-weighted Class Activation Mapping
FNR	False Negative Rate
FP	False Positive
FN	False Negative
TP	True Positive
DINOv2	Self-supervised Vision Transformer Model (Data-efficient Image Transformer v2)
HU	Hounsfield Units
LAD	Left Anterior Descending artery
LCX	Left Circumflex artery
RCA	Right Coronary Artery
AI	Artificial Intelligence
